# Phospholipolysis Caused by Different Types of Bacterial Phospholipases During Cold Storage of Bovine Raw Milk Is Prevented by N_2_ Gas Flushing

**DOI:** 10.3389/fmicb.2018.01307

**Published:** 2018-06-19

**Authors:** Patricia Munsch-Alatossava, Reijo Käkelä, Dominique Ibarra, Mohammed Youbi-Idrissi, Tapani Alatossava

**Affiliations:** ^1^Mikrobiologitoimisto Puustinen & Rahkonen, Helsinki Science Park, Helsinki, Finland; ^2^Molecular and Integrative Biosciences Research Programme, Faculty of Biological and Environmental Sciences, University of Helsinki, Helsinki, Finland; ^3^Air Liquide, Centre de Recherches Paris-Saclay, Jouy-en-Josas, France; ^4^Department of Food and Nutrition, University of Helsinki, Helsinki, Finland

**Keywords:** raw milk, cold storage, N_2_ gas flushing, bacteria, lipidomics, phospholipids, phospholipases (PLases), lysophospholipids

## Abstract

Cold storage aims to preserve the quality and safety of raw milk from farms to dairies; unfortunately, low temperatures also promote the growth of psychrotrophic bacteria, some of which produce heat-stable enzymes that cause spoilage of milk or dairy products. Previously, N_2_ gas flushing of raw milk has demonstrated significant potential as a method to hinder bacterial growth at both laboratory and pilot plant scales. Using a mass spectrometry-based lipidomics approach, we examined the impact of cold storage [at 6°C for up to 7 days, the control condition (C)], on the relative amounts of major phospholipids (phosphatidylethanolamine/PE, phosphatidylcholine/PC, phosphatidylserine/PS, phosphatidylinositol/PI, and sphingomyelin/SM) in three bovine raw milk samples, and compared it to the condition that received additional N_2_ gas flushing (N). As expected, bacterial growth was hindered by the N_2_-based treatment (over 4 log-units lower at day 7) compared to the non-treated control condition. At the end of the cold storage period, the control condition (C7) revealed higher hydrolysis of PC, SM, PE, and PS (the major species reached 27.2, 26.7, 34.6, and 9.9 μM, respectively), compared to the N_2_-flushed samples (N7) (the major species reached 55.6, 35.9, 54.0, and 18.8 μM, respectively). C7 samples also exhibited a three-fold higher phosphatidic acid (PA) content (6.8 μM) and a five-fold higher content (17.3 μM) of lysophospholipids (LPE, LPC, LPS, and LPI) whereas both lysophospholipids and PA remained at their initial levels for 7 days in N7 samples. Taking into consideration the significant phospholipid losses in the controls, the lipid profiling results together with the microbiological data suggest a major role of phospholipase (PLase) C (PLC) in phospholipolysis during cold storage. However, the experimental data also indicate that bacterial sphingomyelinase C, together with PLases PLD and PLA contributed to the degradation of phospholipids present in raw milk as well, and potential contributions from PLB activity cannot be excluded. Altogether, this lipidomics study highlights the beneficial effects of N_2_ flushing treatment on the quality and safety of raw milk through its ability to effectively hinder phospholipolysis during cold storage.

## Introduction

Milk, as the basis of the growth of mammalian progeny, is comprised of water, sugars, proteins, lipids, minerals, and vitamins. By weight, the lipid (or fat) fraction of milk is composed of approximately 98% triacylglycerols, 0.8% phospholipids, 0.3% diacylglycerols, 0.3% sterols, 0.03% monoacylglycerols, and 0.1% free fatty acids, with carotenoids, fat soluble vitamins and flavor compounds present in trace amounts (Taylor and MacGibbon, 2002). Milk lipid biosynthesis occurs inside mammary gland alveolar secretory cells: following lipid synthesis in the endoplasmic reticulum and release into the cytosolic compartment, the lipid droplets migrate to the apical region of the secretory cell where they interact with the plasma membrane. The lipid droplets are then coated with plasma membrane material during the budding phase and are expelled into the alveolar lumen as “milk fat globules" (MFGs) (approximately 1–10 μm in diameter). The membrane structure that surrounds MFGs is called the “milk fat globule membrane" (MFGM), and polar phospholipids as major constituents of cellular membranes are also the major constituents of the MFGM (Keenan and Mather, 2002; Taylor and MacGibbon, 2002). For glycerophospholipids, the sn-1 and sn-2 positions of the glycerol backbone are esterified with fatty acids (of various chain length and degrees of unsaturation), whereas a phosphate group together with an amino-alcohol or sugar group (choline, ethanolamine, serine, or inositol) is attached to the sn-3 position. For sphingolipids, the core amino alcohol called sphingosine is bound to a fatty acyl residue (the ceramide unit), and the addition of a phosphocholine group or of carbohydrates (simple or complex sugars) produces sphingomyelin or glycosphingolipids, respectively.

A recent literature survey highlighted the large variation in the compositions of the most abundant phospholipid classes in milk fats from different mammalian species (Contarini and Povolo, 2013); numerous technical factors such as challenging extraction procedures for separating phospholipids from other milk components, the sensitivity of lipids to oxidation or hydrolysis during sample preparation and the use of different methods for the lipid detection and quantification have likely affected the results. In addition, biological factors such as the cow’s breed and diet and the period of lactation also impact bovine milk lipid profiles (Gallier et al., 2010; Contarini and Povolo, 2013). In bovine raw milk, the relative importance of the different phospholipid classes, expressed as % of total phospholipids, was earlier estimated by Keenan and Mather in 2002: phosphatidylcholine/PC (36%), phosphatidylethanolamine/PE (27%), sphingomyelin/SM (22%), phosphatidylinositol/PI (11%), and phosphatidylserine/PS (4%). Gallier et al. (2010) also reported the presence of phosphatidic acid (PA) (1.8 mol%) in bovine raw milk. For plasma membranes of most eukaryotic cells but also for the MFGM, PC, and SM are predominantly located on the outer leaflet, whereas PE, PS, and PI are mostly located on the inner leaflet (Istivan and Coloe, 2006; Zheng et al., 2014). Due to their amphiphilic structure, phospholipids are of great interest to the food industry as emulsifying agents, as emulsion or foam stabilizers. In addition, phospholipids and their breakdown products are bioactive compounds involved in cell signaling and have been associated with positive effects on heart and brain health, including a reduced risk of neurodegenerative diseases, and antibacterial, anti-inflammatory and anticancer activities, in addition to a protective role in gastro-intestinal mucus (Mac Gibbon and Taylor, 2002; Ehehalt et al., 2010; Contarini and Povolo, 2013; Verardo et al., 2017).

Phospholipases (PLases), that mediate phospholipid catabolism, are diverse and ubiquitous enzymes among eukaryotes or prokaryotes with varied roles: for example, PLases in snake venom trigger lysis of erythrocyte membranes, and PLases also convert phospholipids into lysophosphatidyl derivatives of phospholipids (lysophospholipids) (to initiate lipid digestion).

Depending on the site of action, different classes of PLases (PLAs, PLBs, PLCs, and PLDs) have been defined. PLA1 and PLA2 cleave ester bonds at the sn-1 and sn-2 positions of the glycerol backbone, respectively, producing 2-acyl and 1-acyl lysophospholipids, respectively, together with free fatty acids. The acyl chain that remains at the lysophospholipid molecule can be further targeted by lysophospholipase As (LPLAs). PLBs catalyze the hydrolytic cleavage of both acyl chains located either at sn-1 or sn-2 positions and also display LPLA activity. PLCs target the glycerol-oriented phosphodiester bond and release the phosphorylated head group together with diacylglycerol (DAG). The action of PLDs releases the head group (choline, ethanolamine, serine or inositol) and PA (Istivan and Coloe, 2006; Sitkiewicz et al., 2006; Flores-Diaz et al., 2016). PLases and sphingomyelinases (SMases) are essential virulence factors for a variety of Gram-positive (G+) and Gram-negative (G-) bacteria. As for eukaryotic enzymes, the products of bacterial PLase and SMase hydrolysis act at the membrane level and are involved in alterating cellular signaling pathways, activating the inflammatory cascade and mediating functions supporting growth or leading to cell death (Istivan and Coloe, 2006; Sitkiewicz et al., 2006; Flores-Diaz et al., 2016). Bacterial PLAs and PLBs are either membrane-associated or secreted carboxyl ester hydrolases, some of which trigger haemolytic activity or induce necrosis of the host cells. For example, the highly virulent ExoU marker of *Pseudomonas aeruginosa* possesses PLA2 activity, which disrupts normal host cell signaling functions, induces proinflammatory cytokine production and promotes cytoskeletal collapse and membrane rupture followed by lysis (Sitkiewicz et al., 2006; Sato and Frank, 2014).

Lysophospholipids are considered minor components in foodstuffs (Inoue et al., 2011), including raw milk, where their relative importance is under debate. In one study, lysophosphatidylcholine (LPC) constituted 2% of total phospholipids (Keenan and Mather, 2002); in another study, total LPC and LPE (lysophosphatidylethanolamine) amounts reached 1 and 0.5%, respectively (Gallier et al., 2010), whereas no LPE was detected in raw milk by Sanches-Juanes et al. (2009). Lysophospholipids are either considered artifacts released during handling of cold-stored milk or resulting from phospholipid degradation during analytical extraction procedures, or as hydrolysis products resulting from PLase activity (Gallier et al., 2010). The amphiphilic structure of lysophospholipids also confers surfactant properties which lead to the disruption of lipid–lipid, lipid-protein and protein–protein interactions at the level of the plasma membrane. Similar to phospholipids, lysophospholipids are also of interest to the food industry; for example, lysophospholipids used as exogenous emulsifiers exerted positive effects on the physiological performance and fat digestion of broilers (Polycarpo et al., 2017). Despite their presence in smaller amounts, lysophospholipids are also essential plasma membrane components, and alteration of lysophospholipid contents impacts the spontaneous curvature of membranes and affects the conformation of ion channels (Piñeiro and Falasca, 2012). Further, lysophospholipids serve as intermediate metabolites in phospholipid homeostasis, exhibit hormone-like signaling properties and are involved in the synthesis of various phospholipids and embedding of proteins (Drzazga et al., 2014). Lysophospholipids bind to G protein-coupled receptors (GPCRs) on the surface of several types of immune cells (T and B cells, macrophages, monocytes) (Sitkiewicz et al., 2006). An antibacterial effect is illustrated by the fact that lysophospholipids can directly act on the membranes of G+ bacteria to alter their permeability and damage bacterial integrity (Coonrod and Yoneda, 1983; Arouri and Mouritsen, 2013). Following detection of lysophospholipids in many types of biological samples, they were given roles in multiple physiological or pathophysiological processes (Makide et al., 2014). Both LPC and lysophosphatidylinositol (LPI) play crucial roles as mediators and initiators of varied cellular responses. LPC, the most abundant lysophospholipid of human blood, is a modulator of the inflammation process and was found to have a role in pathological conditions related to atherosclerosis (Drzazga et al., 2014). LPC also belongs to the category of “find-me” signals released by apoptotic cells to attract phagocytes (Gregory and Pound, 2011; Han and Ravichandran, 2011).

Numerous cell types produce LPI, which exerts a stimulatory effect on insulin release by pancreatic islets (Piñeiro and Falasca, 2012). LPI is involved in the regulation of intracellular Ca^2+^ and in cell migration, but it also acts as a mitogen and both LPI and its receptor GPR55 are key regulators of cell proliferation in many cancer types (Falasca and Corda, 1994; Piñeiro and Falasca, 2012). Recently, Woznica et al. (2016) documented the first evidence implicating LPEs in host-microbe interactions when they showed that bacterial lipids, including LPEs, activated or inhibited multicellular development of choanoflagellates (a group of microbial eukaryotes, the closest living relatives of animals).

Raw milk constitutes an ideal growth medium for numerous microbes and the different components of milk also constitute potential substrates for a variety of enzymes. Cold storage, which aims to preserve the quality of raw milk from farms to dairies, does not prevent the growth of psychrotrophic bacteria which produce proteases, lipases and PLases that can degrade milk components and cause spoilage (Chambers, 2002; Dogan and Boor, 2003). Several bacterial genera, well-represented in raw milk, including *Pseudomonas*, *Acinetobacter*, and *Bacillus*, are known to produce spoilage enzymes such as PLC, which disrupt the MFGM, and expose lipids to the endogenous milk lipase or to microbial lipases (Craven and Macauley, 1992). Both *Pseudomonas fluorescens* and *P. aeruginosa* produce a PLC involved in haemolytic activity (Berka and Vasil, 1982; Rossignol et al., 2008). The heat treatments, currently employed in dairies, eliminate most bacteria; however, due to their remarkable heat resistance, many of the secreted bacterial enzymes produced by psychrotrophs (Psy) during cold storage can continue their spoilage actions in different dairy products (Cousin, 1982; Sorhaug and Stepaniak, 1997; Walstra et al., 2006; Machado et al., 2017). A recent study showed that *Pseudomonas*, *Bacillus*, and *Microbacterium* strains, isolated from raw milk, retained 50–75% of their enzymatic activity (either protease, lipase or PLase) after a 142°C/4 s heat treatment (Vithanage et al., 2016).

Strategies for preventing bacterial growth during cold storage should reduce the potential for bacterial spoilage and better preserve the proteins, lipids and phospholipids found in raw milk. Previously, we observed that N_2_ gas flushing of raw milk strongly inhibited bacterial growth at laboratory and pilot plant scales, and Next Generation Sequencing revealed that key spoilers such as *Pseudomonas* were targeted by the N_2_ flushing treatment; earlier studies also suggested that phospholipolytic bacteria present in bovine raw milk were particularly sensitive to N_2_ flushing (Munsch-Alatossava et al., 2010a,b,c, 2016, 2017; Gschwendtner et al., 2016). Moreover, N_2_ flushing treatment conferred a significant protective effect on the antioxidant components of raw milk, during cold storage (Gursoy et al., 2017).

The fact that fewer producers of proteases, lipases or PLases are present in cold-stored while N_2_-flushed raw milk compared to the single cold-stored controls suggests improved preservation of the different components of milk during the storage phase. Using a lipidomics-approach, we undertook to explore the impact of the N_2_ gas flushing treatment on milk phospholipids by comparing bovine raw milk samples that were cold-stored at 6°C for 7 days (the control condition) to samples that were additionally N_2_-flushed. The contribution of different cultivable aerobic bacterial types was simultaneously evaluated.

## Materials and Methods

### Origin and Treatment of Raw Milk Samples

Three independent bovine raw milk samples (M1, M2, and M3) were considered; they represented lorry milk delivered to Helsinki Dairy Ltd. in Helsinki (Finland) at different days; each sample corresponded to pooled raw milk originating from several farms located in Helsinki region. After its reception, the raw milk was dispatched in sterile bottles, placed on a multiplace magnetic stirrer, and continuously mixed while kept at 6.0 ± 0.1°C for 7 days. The continuous N_2_ gas treatment was applied as described previously (Munsch-Alatossava et al., 2010c). Descriptions of the conditions are as follows: C, control; N, N_2_ gas-flushed; 0, day 0; 3, day 3; 7, day 7.

### Microbiological Analyses

The analyses of samples (M1, M2, and M3) were performed shortly after reception of the milk (day 0), and after 3 and 7 days of cold storage, at which times, the N_2_ flushing treatment was shortly interrupted and 0.5 ml raw milk was withdrawn, serially diluted and cultured. The bacterial groups considered and the conditions of the analyses are described in **Table 1**. Production of extracellular enzymes was evaluated by agar diffusion assays, and only distinct bacterial colonies presenting the expected phenotypes on the relevant agars were recorded. All bacterial counts were determined mostly from triplicate (if not duplicate) platings, following aerobic incubation.

**Table 1:** Conditions for the enumeration of the considered bacterial populations present in bovine raw milk samples (M1, M2 and M3) during cold storage for up to 7 days at 6°C.

Bacterial types	Media	Supplier	Incubation conditions	Notes
“Total counts” (TM)	Plate Count Agar (PCA)	Lab M, Ltd., Lancashire, United Kingdom	30°C/3d	All distinct colonies were enumerated
Gram-negative bacteria (G-)	Mac Conkey Agar	”	30°C/3d	All distinct colonies were enumerated
Spores	PCA	”	30°C/3d	Milk samples were heated for 12 min at 80°C prior to the analyses
Protease producers (Pr+)	Skim milk-enriched agar^1^	–	30°C/3d	A clear zone surrounding a colony is indicative of protease activity
Lipase producers (Li+)	Tributyrin + Victoria Blue agar^2^	–	30°C/3d	A dark “blue” zone or a contrasted area that surrounds a colony corresponds to lipase activity
Phospholipase C producers (PLa+)	PCA supplemented with egg yolk^2,3^	–	30°C/3d	A colony surrounded by an opaque ring was considered as producing phospholipase C
Psychrotrophs (Psy)	PCA	Lab M, Ltd., Lancashire, United Kingdom	7°C/10d	All distinct colonies were enumerated
Haemolytic colonies (Hem+)	Blood Agar + 6% sheep blood	bioTrading, Benelux B.V., Mijdrecht, Netherlands)	30°C/3d	Colonies that showed either α or ß-haemolytic activities were recorded^4^

*^1^Modified from Harper et al., 1978; ^2^Dogan and Boor, 2003; ^3^Chrisope et al., 1976;^4^Alexander and Strete, 2001.*

### Mass Spectrometry of Phospholipids

At every sampling time point, 2 × 1.5 ml volumes of raw milk (of samples M1, M2, and M3) were withdrawn and stored at -80°C until analyses. The phospholipid analysis work flow was performed essentially as described by Tigistu-Sahle et al. (2017). Aliquots of milk lipid extracts were evaporated and dissolved in chloroform/methanol (1:2 v/v), spiked with internal standards and supplemented with 1% NH_4_OH just prior to direct infusion of the sample solution, at a flow rate of 10 μl/min, into the ESI source of a triple quadrupole mass spectrometer (Agilent 6490 Triple Quad LC/MS with iFunnel technology, Agilent Technologies, Inc., Santa Clara, CA, United States). In addition to MS+ and MS- scans, MS/MS precursor ion scans were used to detect choline lipid species (PC, LPC, and SM) (precursors for the fragment ion *m/z* 184) and PI species (precursors for *m/z* 241). MS/MS neutral loss scans were applied to detect PE species (neutral loss of 141 amu) and PS species (neutral loss of 87 amu). For the MS analyses, a source temperature of 250°C and instrument collision energies of 5–45 eV were used; the optimal settings were adjusted for each lipid class. Nitrogen served as the nebulizing agent (20 psi) and as the drying gas (11 μl/min at 250°C). The spectra, generated by the instrument, were processed by the Mass Hunter Workstation qualitative analysis software (Agilent Technologies, Inc., Santa Clara, CA, United States), and the individual phospholipid species were quantified using the internal standards and the free software Lipid Mass Spectrum Analysis (LIMSA) (Haimi et al., 2006). Using this software, the spectral peak intensities were converted to concentrations expressed as mole percent (mol%) for each lipid species (relative to the total amount in the lipid class).

### Statistical Analyses

The results, depicted in **Figures 2–5**, are presented as the means ± standard deviation (SD) values from the three raw milk samples (M1, M2, and M3). The data comparison, shown in **Figure 2**, was made by using Student’s *t*-test: the significant difference was marked by asterisks when *p* < 0.01 (^∗∗^). The Pearson’s coefficient (*r*) was used for correlation analysis.

## Results

### Bacterial Populations

**FIGURE 1:**
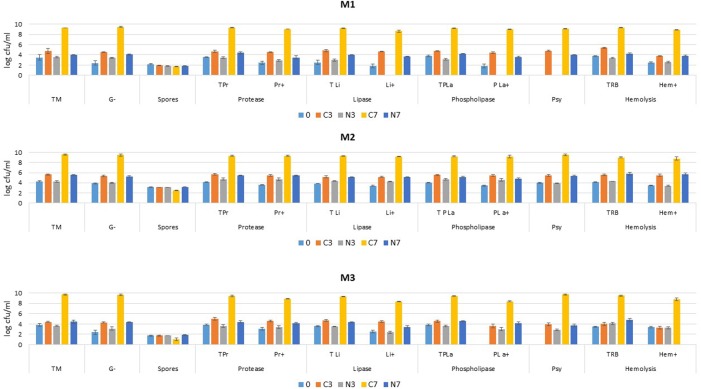
Bacterial counts (log cfu/ml) in bovine raw milk samples (M1, M2, and M3) determined at the initial stage (0) and after cold storage at 6°C for 3 and 7 days (C3, C7) or in cold-stored while N_2_-flushed samples for 3 and 7 days (N3, N7). The bars correspond to standard deviations. TM, “total counts” on PCA; G-, Gram-negative bacteria on Mac Conkey agar; TPr, “total counts” on protease agar; Pr+, protease-positive; TLi, “total counts” on lipase agar; Li+, lipase-positive; TPLa, “total counts” on PLase agar; PLa+, PLase-positive; TRB, “total counts” on blood agar; Hem+, α or ß types of haemolysis; Psy, psychrotrophs.

**Figure 1** shows that initial (day 0) mesophilic “total counts” (TM) ranged between 3.2 and 4.2 log-units for samples M1, M2, and M3. Initial G-bacteria were less numerous in samples M1/0 and M3/0 compared to M2/0, as they represented 8, 39, and 4% of TM counts in samples M1, M2, and M3, respectively. Spore levels were not impacted by the applied conditions in any of the considered samples (M1, M2, and M3).

Initial TM enumerated on the protease, lipase, and PLase agar types (TPr, TLi, and TPLa) mostly reflected initial TM counts. Bacterial enzymatic activities were initially detected in all three samples, except for M3/0 where no PLase producers (PLa+) were identified at day 0. Psy were already enumerated at day 0 for sample M2 at a level equivalent to TM, whereas Psy were only detected in samples M1 and M3 after 3 days of cold storage, at which stage the level was also equivalent to TM. Initial “total counts” on blood agar (TRB) were close to TM counts recorded on PCA agar, whereas initial haemolytic colonies (Hem+) were below the corresponding “total counts” (TRB) for M1 and M2, except for M3 where all colonies displayed haemolytic features.

For the control raw milk samples, the TM counts in general moderately increased during the first 3 days of the cold storage period (C3), but substantially rose in the following days (C7). The dynamics of growth was greater for M2/C3, as counts already exceeded 10^5^ cfu/ml after 3 days, whereas a slower rise kept counts below the 10^5^ level for M1/C3 and M3/C3. For all three samples, the colonies that exhibited protease, lipase and PLase activities (Pr+, Li+, and PLa+) in the controls (C3) were at about the same levels at day 3 as the TM enumerated on the corresponding agar types. Between 3 and 7 days, the rise of proteolytic, lipolytic or phospholipolytic bacteria (Pr+, Li+, and PLa+) continued and equalled TM (TPr, TLi, and TPLa) of the corresponding controls for M2/C7, but were moderately outnumbered by TM for M1/C7 and M3/C7. The number of colonies recovered on blood agar (TRB) for the controls (C3) also reflected TM counts; haemolytic colonies (Hem+) remained constant for M3/C3 and increased moderately for M1/C3, but showed the greatest increase for M2/C3 during the initial cold storage phase. The additional 4 days of cold storage promoted a considerable increase in haemolytic colonies (Hem+) in all three samples.

Combining cold storage with N_2_ flushing prevented an increase in TM in all three samples at 3 days. The treatment also hindered an increase in G-bacteria for M2/N3, though a moderate increase in G-bacteria was observed for M1/N3 and M3/N3. Nevertheless, all counts recorded for the N_2_-flushed milk samples (N3) were below the counts recorded for the corresponding controls (C3).

After 7 days of cold storage, whereas TM had increased by approximately 1.5 log-units for M2, it remained at roughly its initial level for samples M1 and M3 and was still below the 10^5^ cfu/ml (5 log units) critical level. Under N_2_, G-bacterial counts increased constantly but moderately between 0 and 7 days for M1 and M3; in M2, during the first 3 days, the treatment did not impact the amount of G- bacteria, which later increased moderately between 3 and 7 days. In the N_2_-flushed milk samples after 7 days (N7), for the most part, as with TM, the levels of G- bacteria attained the levels recorded for the control samples at 3 days (C3).

The N_2_ flushing treatment affected TPr, TLi, and TPLa counts similarly, as all were equivalent to TM for M1/N3 and M3/N3 at day 3, or showed a slight increase for M2/N3. At day 3, the comparison of Pr+, Li+, and PLa+ counts with the corresponding “total counts” (TPr, TLi, and TPLa) revealed contrasted situations. For M2/N3, the colonies that produced enzymes were as numerous as the corresponding “total counts”, but for M3/N3 if Pr+ were at the same level than TPr, Li+, and PLa+ counts were below the corresponding “total counts” (TLi, TPLa). For M1/N3, Pr+ counts were below the TPr level, and no lipase or PLase producers (Li+, PLa+) were detected at day 3. The additional 4 days of cold storage showed the most modest impact on M2/N7 for Pr+ and Li+ levels, which moderately increased, whereas the level of PLa+ remained about constant between 3 and 7 days. The counts for M2/N7 were very similar to those for M2/C3. For M1/N7 at day 7, Li+ and PLa+ colonies were again detected, while Pr+ were slightly below the TPr level. The sample M3/N7 was characterized by a moderate increase in enzyme producers equivalent to TPr, TLi, and TPLa levels for protease and PLase producers, but lower for lipolytic colonies.

Concerning the Psy, N_2_ flushing revealed a contrasted situation between the raw milk samples. In M2, the TM and Psy levels were equivalent along the experiment, whereas in samples M3 and M1, Psy colonies were only enumerated after 3 and 7 days, respectively. At the end of the storage period, Psy and TM levels were also equivalent in M1 and M3.

The N_2_ flushing treatment hindered the rise of TRB bacteria to a degree similar to that of TM and halted the growth of Hem+ colonies during the first 3 days in all three samples. In the following days, the levels of Hem+ colonies for M1/N7 and M2/N7 corresponded to those recorded for the controls M1/C3 and M2/C3 at day 3, whereas Hem+ colonies were below detection level after 7 days of N_2_ flushing for M3/N7.

### Phospholipid Levels and Classes

**FIGURE 2:**
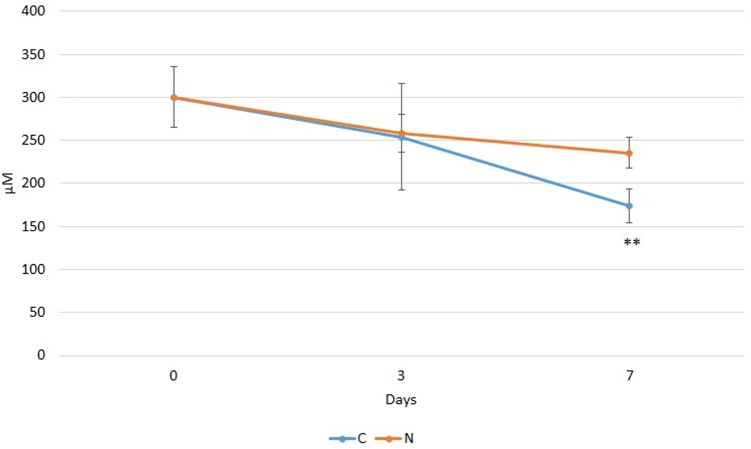
Trends in total phospholipid concentrations (in μM), over time detected in raw milk samples (M1, M2, and M3) stored at 6°C (C), or additionally flushed with N_2_ (N). Note: The mean values (*n* = 3) are significantly different between C7 and N7 (*p* < 0.01) (^∗∗^).

As indicated in **Figure 2**, an average concentration of 300 ± 35 μM phospholipids (approximately 23 mg/100 ml of raw milk) was recorded at day 0 for the three considered raw milk samples (M1, M2, and M3). After 3 days of cold storage, the average phospholipid concentrations had dropped for both C3 and N3, though their levels were not significantly different from the average initial value. In contrast, at day 7, the phospholipid concentration was significantly lower (*p* < 0.01) for C7 (172 μM) compared to N7 (236 μM), though there was still no significant difference between the levels in N7 and at day 0. The Pearson’s correlation coefficient (*r*), calculated for the differences in PLa+ counts (in log units) and the total phospholipid concentration differences (in μM) between controls and N_2_-treated samples, was 0.88.

**FIGURE 3:**
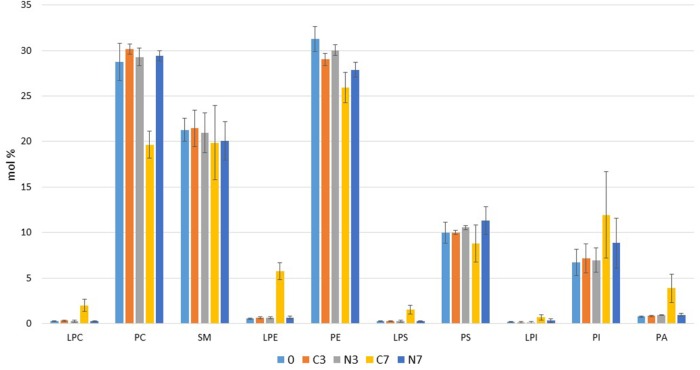
Profiles of phospholipid classes detected in bovine raw milk samples (M1, M2, and M3) (in mol%) at the initial stage (0) and after 3 and 7 days in cold-stored (C3, C7) and in cold-stored while N_2_-flushed (N3, N7) conditions. LPC, lysophosphatidylcholine; PC, phosphatidylcholine; SM, sphingomyelin; LPE, lysophosphatidylethanolamine; PE, phosphatidylethanolamine; LPS, lysosphosphatidylserine; PS, phosphatidylserine; LPI, lysophosphatidylinositol; PI, phosphatidylinositol; PA, phosphatidic acid.

Class level phospholipid profiling revealed that SM, PE, and PS levels which were initially, around 22, above 30 and 10 mol%, respectively, remained altogether quite constant for 3 days in the cold-stored raw milk samples with or without N_2_ flushing (**Figure 3**). In the control condition (C7), a minor decrease was recorded for PS, and a moderate decrease was also observed for PE. On the other hand, PC (initially approximately 28 mol%) showed a major reduction by over 10 mol%, compared to all other conditions (0, C3, N3, and N7). At the initial stage, PI levels were approximately 7 mol%, and by day 7 (C7), they had increased approximately 5 mol%, though this rise was characterized by large sample variation.

Concomitantly, in C7, levels of the three lysophospholipids [LPC, LPE, and LPS (lysosphosphatidylserine)] increased to approximately 2.0, 6.0, and 1.5 mol% of total phospholipids, respectively, whereas the relative amounts remained at their initial levels in N7. LPI levels had only slightly increased by day 7 in the control samples (C7). Cold storage for 7 days also resulted in a slight increase in PA levels to approximately 4 mol%, for the control samples (C7) only.

### Sphingomyelin Species

**FIGURE 4:**
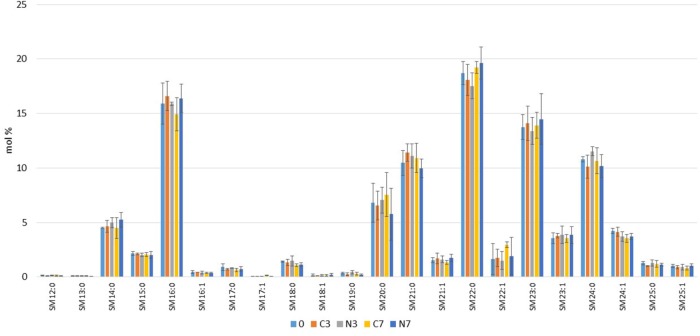
Comparison of molecular species of sphingomyelins (SMs) detected in bovine raw milk samples (M1, M2, and M3) (in mol%) at the initial stage (0) and after 3 and 7 days in cold-stored while N_2_-flushed milk (N3, N7) compared to the only cold-stored controls (C3, C7).

As detailed in **Figure 4**, irrespective of the conditions (control or N_2_-treated milk) or the stage of the cold storage, the analyses showed constant relative levels of both the dominant SM species (16:0, 21:0, 22:0, 23:0, and 24:0, all with the sphingosine backbone), which each exceeded 10 mol% of the total SMs present in the samples at day 0, and the species (14:0, 15:0, 16:1; 17:0; 18:0; 20:0; 21:1; 22:1; 23:1; 24:1; 25:0, and 25:1), which ranged between 0.5 and 10 mol%. The SMs at the limit of detection (12:0, 13:0, 17:1, 18:1, and 19:0) also remained constant throughout the experiments.

### Glycerophospholipid Species

**FIGURE 5:**
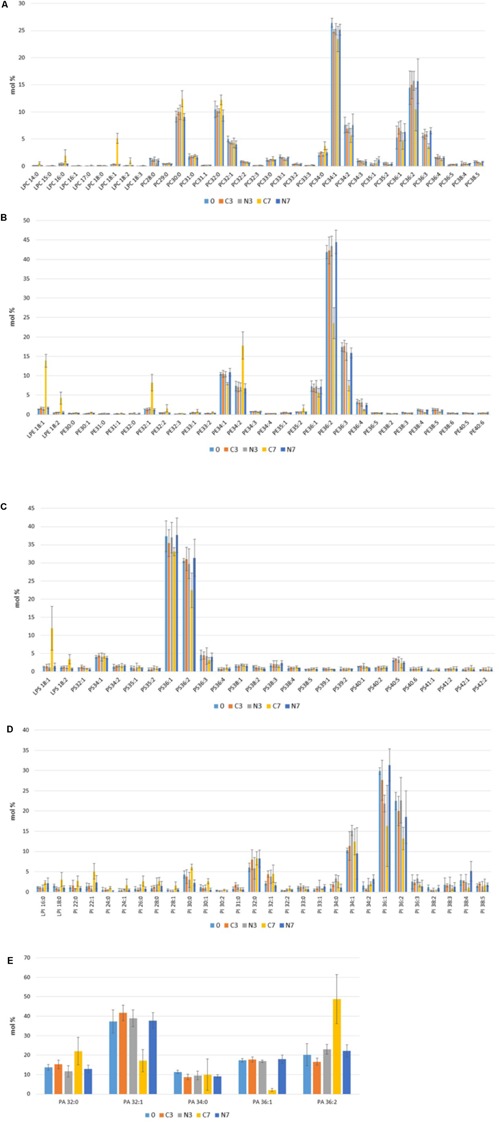
Molecular species of **(A)** phosphatidylcholine (PC), **(B)** phosphatidylethanolamine (PE), **(C)** phosphatidylserine (PS), **(D)** phosphatidylinositol (PI) and **(E)** phosphatidic acid (PA) detected in bovine raw milk samples (M1, M2, and M3) (in mol%) at the initial stage (0) and after 3 and 7 days in cold- stored while N_2_-flushed milk (N3, N7) compared to the only cold-stored controls (C3, C7).

Among the PC species, the most abundant 34:1 and 36:2 were present at relatively constant levels over time, except for a decrease in 36:2 observed between 3 and 7 days in the controls (C7) (**Figure 5A**). The PC species, ranging between 5 and 12 mol%, included saturated species 30:0 and 32:0 (for which the levels recorded for C7 were moderately higher compared to initial levels and to N7) and unsaturated species 34:2, 36:1, and 36:3 (for which the C7 levels were below the initial levels but also below the corresponding N7 levels). Both saturated and unsaturated PC species, which represented less than 2.5 mol% each (28:0, 29:0, 31:0, 32:2, 33:0, 33:1, 33:2, 34:3, 35:1, 35:2, 36:4, 38:4, and 38:5), were present at similar levels, irrespective of the conditions or the time (either initial, intermediate or final stages of the storage period) apart from a moderate increase in PC 34:0 observed in C7. Over the course of the experiments, several PC species were present at the limit of the detection level. The control condition C7 showed an increase in levels of LPC 18:1 and to a lesser extent in LPC 16:0, 18:2, and 14:0, which reached 5, 1.5, 1, and 0.5 mol% of the total PCs species, respectively. In contrast, these LPC species remained at their initial levels in the N_2_-flushed milk samples.

The PE species profile was overwhelmingly dominated by the unsaturated molecular species 36:2, which represented over 40 mol% of PEs at the initial stage, followed by 36:3 (at approximately 18 mol%). For the control conditions (C7), these species showed a remarkable drop after 7 days of cold storage, in contrast to N7 (**Figure 5B**). Several other unsaturated PE species (34:1, 36:1, 36:4, 38:4, and 38:5), present initially at relative levels ranging from 2 to 10 mol%, remained constant during the N_2_ flushing treatment (N7), whereas in the control conditions (C7), these species showed moderate drops in comparison to their initial levels (0). In addition, PE 32:1 and especially 34:2 increased by approximately 7 and 10 mol%, respectively, during the 7-day cold-storage period in C7, unlike N7, for which both species remained at their initial levels. In C7, slight increases were recorded for the minor molecular species 32:2, 33:1, 33:2, and 35:2. The analyses also highlighted a considerable increase in LPE 18:1 (of approximately 12 mol%), and a smaller increase in LPE 18:2 (of approximately 3 mol%) after 7 days of cold storage in the control raw milk samples (C7). In contrast, the PEs and LPEs levels recorded from the N_2_-flushed samples (N3 and N7) remained remarkably constant during the experiments, as their initial composition and levels were mostly preserved.

Among the PS species, the two dominant unsaturated species (36:1 and 36:2) represented 37 and 30 mol%, respectively, of the total PSs species initially present in raw milk (**Figure 5C**). When compared to days 0 and 3, a drop was recorded for PS 36:2 in the control condition (C7). There was little variation between the applied conditions in levels of the three PS species (34:1, 36:3, and 40:5), that were above 2 mol% each; this observation was also valid for the numerous species that were present at trace amounts. The control condition (C7) was also characterized by an increase in LPS 18:1 and LPS 18:2 of approximately 12 and 4 mol%, respectively. In contrast to the controls (C7), the N_2_-flushed raw milk samples (N7) did not show any changes in PS composition or levels, which both remained constant for 7 days.

The PI species, 36:1 and 36:2 initially dominated in the raw milk samples reaching 30 and 23 mol%, respectively (**Figure 5D**). PI 32:0 and 34:1 were within the range of 5–10 mol%, whereas PI 30:0, 32:1, 36:3, and 38:4 ranged between 2.5 and 4 mol%, followed by PI 22.0, 22:1, 30:1, 33:0, 34:2, 38:2, 38:3, and 38:5 which had values between 1 and 2 mol% at the initial stage. Ten other PI types were present at very low levels, and the two lyso-species LPI 16:0 and 18:0 were initially at approximately 1 mol%. Extended cold storage promoted an important drop (by about half of their initial levels) of the dominant species PI 36:1 and 36:2 in C7. Despite large variations among the three raw milk samples, the N_2_ gas treatment (N7) better preserved PI levels over time.

Though the levels of most of the minor PI species (24:0, 31:0, 33:0, 33:1, 34:2, 36:3, 38:3, and 38:5) remained constant in the control conditions (C3, C7), some increases were recorded in C7 for PI 22:0, 22:1, 24:1, 26:0, 28:0, 28:1, 30:0, 30:1, 32:0, 32:1, 32.2, 34:0, and 34:1. The flushing treatment (N7) resulted in a slight increase in PI 32:0, 34:2, 38:4 and a slight decrease in 30:0 and 36:3, compared to their initial levels, though these results were characterized by some sample variation. Concerning lysophospholipids, a moderate increase was recorded for LPI 16:0, irrespective of the applied treatment (C7 or N7), whereas N_2_ gas flushing (N7) prevented an increase of LPI 18:0, contrarily to the observations from the only cold-stored samples (C7).

The five PA species detected were dominated by PA 32:1, which was initially present at 38 mol%, followed by the species 36:2 and 36:1 at 20 and 19 mol%, respectively. PA 32:0 and 34:0 each constituted over 10 mol% of the total detected PA species (**Figure 5E**). Irrespective of the conditions, PA 34:0 levels showed remarkable stability over time for C7, though large sample variation was associated with this result. In the control condition (C7), the 7-day cold storage period promoted a considerable increase of almost 30 mol% for PA 36:2, and an increase of approximately 10 mol% for PA 32:0 (with large sample variations); at the same time, decreases of approximately 20 and 16 mol% were recorded for PA 32:1 and 36:1, respectively. The N_2_ flushing treatment did not impact PA levels over time.

Differences in total phospholipid concentrations between C7 and N7 (**Figure 2**) are detailed for the major phospholipid species in **Table 2**. The total phospholipid concentrations, which were approximately 155 and 232 μM for C7 and N7, respectively, indicate that phospholipids were better preserved in the N_2_-flushed milk samples (N7). This point is further illustrated by the fact that the concentrations of PC, SM, PE, and PS classes were respectively, 48, 27, 18, and 35% lower for C7 compared to N7. Considering the major PCs, PEs, PSs, PIs, and SMs species, with the exceptions of PE 34:1, PE 34:2, and PI 34:1, all phospholipid concentrations recorded for N7 exceeded the corresponding values of C7, and for the species PC 34:1, 34:2, 36:1, 36:2, 36.3, PE 36:2, 36:3, PS 36:2, and PI 36:1, concentrations in N7 were over two-fold higher than in C7 (**Table 2**).

**Table 2:** Major differences in concentration (μM) among phospholipid (PC, SM, PE, PS, PI, and PA) and lysophospholipid (LPC, LPE, LPS, and LPI) species present in bovine raw milk samples (M1, M2, and M3) that were either cold-stored at 6° C for 7 days (C7) or additionally flushed with N_2_ (N7).

Phospholipid classes and species	C7 (*n* = 3) μM	N7 (*n* = 3) μM	C7-N7 μM^a^	PROPOSED PLases or SMases	
	
				Lipidomics data	Microbiological data
***PC class***					
Major LPCs					
14:0	0.23	0.04	+0.18	PLA, PLB	
16:0	0.76	0.27	+0.49	PLA, PLB	
18:1	1.98	0.18	+1.80	PLA, PLB	
18:2	0.40	0.04	+0.36	PLA, PLB	
Major PCs	*27.21*	*55.60*			
30:0	4.77	6.34	-1.57	PLA, PLB	PLC^d^
32:0	4.63	6.57	-1.93	PLA, PLB	PLC
34:1	8.82	17.59	-8.77	PLA, PLB	PLC
34:2	2.20	5.44	-3.24	PLA, PLB	PLC
36:1	1.71	4.32	-2.61	PLA, PLB	PLC
36:2	3.73	10.75	-7.02	PLA, PLB	PLC
36:3	1.35	4.59	-3.24	PLA, PLB	PLC
*All LPCs+PCs^b^*	36.13	70.06	-33.93 (-48%^c^)	PLA, PLB	PLC
***SM class***					
LSMs	N.D.^e^	N.D.	–		
Cer-1-P	N.D.^e^	N.D.	–		
Major SMs	*26.67*	*35.85*			
16:0	5.13	7.69	-2.56	SMase C	
20:0	2.61	2.70	-0.09	SMase C	
21:0	3.82	4.68	-0.86	SMase C	
22:0	6.61	9.22	-2.61	SMase C	
23:0	4.81	6.79	-1.98	SMase C	
24:0	3.69	4.77	-1.08	SMase C	
*All SMs^b^*	34.42	47.02	-12.60 (-27%^c^)	SMase C	
***PE class***					
Major LPEs					
18:1	7.74	1.17	+6.57	PLA, PLB	
18:2	2.34	0.40	+1.93	PLA, PLB	
Major PEs	*34.59*	*53.97*			
34:1	4.41	4.07	+0.34	PLA, PLB	PLC
34:2	10.03	4.59	+5.44	PLA, PLB	PLC
36:1	3.01	4.77	-1.75	PLA, PLB	PLC
36:2	13.05	29.79	-16.74	PLA, PLB	PLC
36:3	4.09	10.75	-6.66	PLA, PLB	PLC
*All LPEs+PEs^b^*	55.26	67.27	-12.01 (-18%^c^)	PLA, PLB	PLC
***PS class***					
Major LPSs					
18:1	2.02	0.40	+1.62	PLA, PLB	
18:2	0.63	0.22	+0.40	PLA, PLB	
Major PSs	*9.94*	*18.80*			
36:1	5.89	10.21	-4.32	PLA, PLB	
36:2	4.05	8.59	-4.54	PLA, PLB	
*All LPSs+PSs^b^*	17.77	27.18	-9.40 (-35%^c^)	PLA, PLB	
***PI class***					
Major LPIs					
16:0	0.54	0.54	+0.00	PLA, PLB	
18:0	0.67	0.27	+0.40	PLA, PLB	
Major PIs	*8.59*	*12.82*			
34:1	2.84	2.11	+0.72	PLA, PLB	
36:1	3.01	7.02	-4.00	PLA, PLB	
36:2	2.74	3.69	-0.94	PLA, PLB	
*All LPIs+PIs^b^*	21.69	21.87	-0.18 (-1%^c^)	PLA, PLB	
***PA class***					
LPAs	N.D.^e^	N.D.	–		
Major PAs	*6.74*	*2.27*			
32:0	1.53	0.31	+1.21	PLD	
32:1	1.21	0.85	+0.36	PLD	
34:0	0.72	0.22	+0.49	PLD	
36:1	0.13	0.40	-0.27	PLD	
36:2	3.15	0.49	+2.65	PLD	
*All PAs^b^*	6.79	2.29	+4.50	PLD	
**Summary:**					
LPLs	17.31	3.53	+13.78	PLA, PLB	
PLs	154.75	232.16	-77.41	SMase C	PLC^d^
LPLs + PLs	172.06	235.69	-63.63 (-27%)^c^	PLA, PLB, PLD, SMase C	PLC
PAs	6.79	2.29	+4.50	PLD	

*^a^Negative versus positive values correspond to decrease versus increase of phospholipid concentrations in μM. ^b^“All” refers to the total phospholipids and lysophospholipids recorded for the considered phospholipid class. ^c^The difference between C7 and N7 is expressed as %. ^d^A PLC activity is proposed for PCs and PEs considering that the PC and PE classes are dominant among egg yolk phospholipids (Zhao et al., 2011); egg yolk agar was used to enumerate PLa+ colonies (see **Table 1**). ^e^N.D., not detected; LSMs, lysosphingomyelins; Cer-1-P, Ceramide-1-Phosphate; LPA, lysophosphatidic acid. The PLases (PLA, PLB, PLC, and PLD) and SMases (SMase C) activities, which could explain the recorded concentration differences, based on the lipidomics and microbiological data, are also indicated.*

Simultaneously and conversely, higher lysophospholipid concentrations (up to 10-fold for LPC 18:1 and 18:2, and up to five-fold for LPE 18:1, 18:2, and LPS 18:1) were observed for C7 compared to N7. With the exception of PI 36:1, which was higher in N7, the fewest changes were detected in the PI class, as LPIs and PIs species concentrations did not greatly diverge between C7 and N7. Except for PA 36:1, which was at a slightly higher concentration in N7, the concentrations of the other four PA species detected were slightly higher in C7 (**Table 2**).

The massive increase in PLase C producers (PLa+) by 10^4^ – to over 10^6^-fold in C7 for the samples M1, M2, and M3 (**Figure 1**) supports the view that the phospholipid hydrolysis mostly resulted from bacterial PLC activity. Higher PA species contents in C7 may be due to enhanced PLD activity. Higher total lysophospholipid concentrations for C7 (17.3 μM) compared to N7 (3.5 μM) may result from PLA and PLB activities, whereas the hydrolysis of SMs species can be attributed to SMase C activity.

## Discussion

For bovine raw milk, bacteriological quality criteria stipulates that the cultivable mesophilic (aerobic/facultative anaerobic) bacterial population fraction should not exceed 10^5^ and 3 × 10^5^ cfu/ml at the farm tank and at the dairy silo, respectively (Anonymous, 2004). As initial TM were approximately 10^4^ cfu/ml (**Figure 1**), the three raw milk samples analyzed here (M1, M2, and M3), were considered of very good bacteriological quality. Higher initial G- bacterial and Psy levels for sample M2 suggest that this raw milk underwent a longer period of cold storage prior to analysis than M1 and M3. In the controls (C), the analyses of the contribution of the cultivable aerobic and facultative anaerobic bacterial population fractions showed that cold storage at 6°C alone promoted bacterial growth in a sample-dependent manner. During cold storage, the increases in TM and in G- bacteria levels were roughly equivalent and coincided with a rise in spoilage enzyme producers (Pr+, Li+, and PLa+) (**Figure 1**), which is in agreement with previous studies (Cousin, 1982; Sorhaug and Stepaniak, 1997; Chambers, 2002). The previously described inhibitory effect of N_2_ gas flushing on bacterial growth (Murray et al., 1983; Dechemi et al., 2005; Munsch-Alatossava et al., 2010a,b,c, 2017, 2016; Gschwendtner et al., 2016) was also observed here. Cold storage combined with N_2_ gas flushing prevented all considered bacterial types, including exoenzyme producers from exceeding 10^5^ cfu/ml for samples M1 and M3 over the course of 7 days. The inhibitory effect was probably attenuated for M2 due to a higher initial bacterial load. Taking into consideration both the initial TM and the times required to reach a 10^5^ cfu/ml bacterial content (**Figure 1**), the efficiency we observed for the effect of the N_2_ treatment in M2 compared to M1 and M3 suggests that N_2_ flushing should be applied at the earliest possible time point, when bacterial counts are minimal.

In principle, both PLA and PLC activities are detectable on lecithin or egg yolk agar: on the agar plates turbid zones correspond to PLC activity, whereas PLA production results in clear zones surrounding bacterial colonies (Chrisope et al., 1976; Titball, 1993). In this study, the egg yolk agar was used to enumerate cultivable PLC producers (PLa+, in **Figure 1**); clearly, PLa+ bacterial types, which were considerably increased in the controls (C7), were affected by the N_2_-treatment, but in contrast to previous observations (Munsch-Alatossava et al., 2010a), the 7 days of N_2_ flushing did not restrict the numbers of PLC producers to below detection level in the samples tested (**Figure 1**). In addition to PLases C or SMases C activities, other secreted bacterial factors (considered as pathogenic factors) including cyclic lipodepsipeptides produced by *Pseudomonas* for example, can also provoke haemolysis (Rainey et al., 1991; Munsch and Alatossava, 2002). For the three tested raw milk samples, the observation that the PLa+ and Hem+ bacterial counts did not exactly coincide, despite similar TPLa and TRB counts (**Figure 1**), suggests that the detected haemolytic responses were caused by various factors. The extent of haemolytic responses, evaluated here with agar containing 6% sheep blood cells, may be still underestimated, as Fiester et al. (2016) demonstrated that *Acinetobacter baumanii* (also found in raw milk, Munsch-Alatossava and Alatossava, 2006; Gschwendtner et al., 2016; Vithanage et al., 2016) was poorly hemolytic to sheep erythrocytes.

During cold storage, a minor fraction of milk fat adsorbed to the walls of the glass bottles in both controls and N_2_-treated samples, most likely leading to a moderate decline in detected phospholipid levels for raw milk samples incubated for 3–7 days (**Figure 2**). For the three raw milk samples, the average recorded initial phospholipid concentration was 300 μM (**Figure 2**), equivalent to approximately 23 mg/100 ml of raw milk, which is in line with the study by Garcia et al. (2012) that reported 20.4 mg phospholipids/100 ml of cow milk.

Our results for initial phospholipid class composition of the three raw milk samples (PE, PC, SM, PS, and PI at 30, 28, 20, 10, and 6 mol%, respectively) (**Figure 3**), agreed better (except for PS) with the data obtained by Christie et al. (1987) (in weight %, PE 34.2%, PC 25.4%, SM 23.6%, PS 2.9%, and PI 6.2%) than with the data by Keenan and Mather (2002). PA, rarely detected in raw milk (Verardo et al., 2017), corresponded at the initial stage (day 0) (**Figure 5E**) to the level determined by Gallier et al. (2010). Initial lysophospholipid levels were at the limit of detection for LPC, LPS, and LPI, and at approximately 1% for LPE (**Figures 3, 5**): these results are consistent with previous studies (Keenan and Mather, 2002; Gallier et al., 2010).

Freezing of milk samples may cause physicochemical changes to the lipoprotein complexes, and physical damage caused by ice crystals can damage the MFGM (Fox, 2002). However, the levels detected here, of less than 1 mol% for some phospholipids including lysophospholipids (**Figures 4, 5**), which showed remarkable consistency in some conditions over time, together with the fact that both N_2_-treated and control raw milk samples were simultaneously analyzed, eliminates the risk that the major recorded phospholipid composition changes may result from sample storage or handling, or the applied experimental procedure.

Altogether, phospholipid composition profiles were similar for most species at the initial and intermediate stages of the experiments for both controls (C) and N_2_-treated raw milk samples (N). The N_2_ flushing treatment (N7) preserved still the initial phospholipid composition during the additional 4 days of cold storage (**Figures 3, 5**). In contrast, the single 7 days-cold storage condition (C7) promoted a drop in phospholipids concomitant with an elevation in lysophospholipids (LPC, LPS, LPI, and mostly LPE) (**Figures 3, 5** and **Table 2**). The major drops in PC 36:2 and especially in PE 36:2 species (approximately 17 mol%) coincided with higher levels of LPE 18:1, LPS 18:1, LPC 18:1, LPE 18:2, LPS 18:2, LPI 18:0, and LPC 16:0 which reached 13, 12, 5, 4, 4, 3, and 2 mol % among each phospholipid class, respectively (**Figures 3, 5**).

Initial SMs were approximately 23 mol% here, which is in line with the studies by Christie et al. (1987) and Keenan and Mather (2002) that reported relative amounts of 23 and 22% of SMs (of total phospholipids), respectively. The report by Mac Gibbon and Taylor (2002) listed SM 16:0, 22:0, 23:0, and 24:0 as major SM species in bovine milk representing 22.9, 15.2, 28.0, and 15.4% respectively; similarly, here, the same SM species were preponderant, however, they varied in amounts, as SM 16:0, 22:0, 23:0, and 24:0 accounted for 15, 18, 14, and 10 mol% of the total SMs respectively (**Figure 4**). In addition, SM 21:0, which was not detected in the previous study, reached 10 mol% in our investigations (**Figure 4**).

N_2_ flushing also seemed to better preserve SMs composition in raw milk during cold storage, as the total concentration of SMs for N7 was 27% greater than that of C7 (**Table 2**). Bacterial SMases C, which cleave the ester bond between ceramide and phosphorylcholine, are cytotoxic to certain cells and haemolytic for certain species. The production of SMase Cs was demonstrated in *Bacillus*, *Listeria*, and *Pseudomonas* genera (Flores-Diaz et al., 2016), which are all commonly found in raw milk. The drop in SMs concentration in the controls (**Table 2**) can reasonably be attributed to SMase Cs synthesized by rising bacterial populations (**Figure 1**). It was reported that SMase Ds hydrolyse LPC, which generates lysophosphatidic acid (LPA) (Flores-Diaz et al., 2016); the absence of LPA together with the presence of various lysophospholipids (**Figures 3–5** and **Table 2**) suggest low, if any, SMase Ds or lysophospholipases D activities in the raw milk samples tested. For all metabolites, the recorded concentrations reflect the balance between synthesis and degradation; consequently, the presence of some phospholipid and lysophospholipid species at the limit of detection together with the absence of other species should be interpreted with care, as for example, PLC can degrade LPI (Drzazga et al., 2014) and lysophospholipase D can convert LPI to LPA (Piñeiro and Falasca, 2012).

Compared to N7, the controls (C7) revealed higher PA contents (**Figures 3, 5E** and **Table 2**). PA, which results from PLD activity, exerts multiple roles as a signaling molecule and modulator of enzymatic activity, but it also promotes negative curvature of membranes (Flores-Diaz et al., 2016). The changes observed may also be linked to enzymatic production during bacterial growth, as it has been shown that the genomes of *A. baumanii* or *P. aeruginosa* encode up to three and two different PLDs, respectively (Russell et al., 2013; Stahl et al., 2015).

High hydrolysis rates of certain phospholipids may be related not only to their relative abundance in raw milk but also to their respective locations at the MFGM level. For example, PC and SM are predominantly situated at the outer leaflet (Zheng et al., 2014) and are well- exposed to enzymatic degradation.

The analyses considered two sampling points (days 3 and 7) that enabled us to determine the raw milk’s phospholipid composition with a “total” bacterial content (TM) below 10^5^ cfu/ml (at day 3, for M1 and M3) or above 10^5^ cfu/ml at day 7 in all controls. The results from the time point “day 7” (where samples exhibited “total bacterial counts” far above 10^5^ cfu/ml) allowed us to visualize phospholipid composition in “extreme situations.” The extent of phospholipolysis recorded here, together with the fact that for example in Australia raw milk is mostly kept in refrigerated conditions for 3–5 days in the farm bulk tank before being delivered to dairy processing plants (Vithanage et al., 2016), calls for further investigation into phospholipid composition at more frequent sampling points.

Cold storage selects for psychrotrophic bacterial types that produce a remarkably diverse enzymatic arsenal which is mainly considered under its spoilage potential. Considering PLases, it is well known that cold storage of raw milk promotes enhanced PLC activity resulting from psychrotrophic bacterial contaminants (Craven and Macauley, 1992; Dogan and Boor, 2003; Vithanage et al., 2016). Also in this study, based on microbiological analyses, PLCs appeared as a major contributor to phospholipolysis during cold storage considering that the totality (for M1 and M2) or the majority (for M3) of the bacterial populations recovered from PLase agar expressed a PLa+ phenotype (**Figure 1**).

As changes in phospholipid composition (**Figures 2–5**) coincided with increased bacterial levels in controls (**Figure 1**), and as no or minor changes in phospholipid composition coincided with bacterial growth inhibition by N_2_ flushing (**Figure 1**), combined with the fact that some hydrolysis products (e.g., lysophospholipids and PA) result from specific PLases activities, we may attribute the detected changes to an increase in production of varied PLases, PLC, PLA, PLB, PLD, and SMase C production by rising bacterial populations; this point is corroborated by the high value of the Pearson’s correlation coefficient that indicated a strong positive relationship between PLa+ and phospholipid concentration differences.

The elevated lysophospholipid content in C7 (compared to the initial stage) together with the fact that only PLB exhibits LPLAs activity leading to the degradation of lysophospholipids, suggests that PLA could be predominant over PLB. With the hypothesis of minor or negligible PLB activity, the enzymatic activities responsible for total phospholipolysis that occurred in raw milk during the single cold storage may be roughly estimated to account here for approximately 80, 15, and 5% for PLC together with SMase C, PLA, and PLD, respectively (**Table 2** and **Figures 1, 2**).

To the best of our knowledge, no studies have yet revealed an increase in lysophospholipids to this extent in cold-stored raw milk. Nevertheless, the impact of cold storage on phospholipidic composition in food has been previously reported for beef meat stored at 4°C for 14 days, which showed a continuous increase in LPE and LPC levels consequent to PLA2 activity (Rauch and Kaš, 1983). The evaluation of the possible consequences of the presence of higher amounts of lysophospholipids in cold-stored raw milk should take into consideration both the nature of the lysophospholipids released during cold storage as well as their levels, as lysophospholipids, which have a greater stability than phospholipids (Polycarpo et al., 2017), will likely persist in heat-treated milk. The variable metabolic fate of lysophospholipids in mammals was investigated by Inoue et al. (2011), who demonstrated a selective protection of lysophospholipids from metabolism and absorption by the rat digestive system: LPA was absorbed and metabolized to a lesser extent in the digestive tract compared to LPC and LPE. In addition, higher lysophospholipid levels in the intestinal tract may impact gut microbiota diversity: as previously shown, lysophospholipids have an antibacterial effect on G+ bacteria (Coonrod and Yoneda, 1983; Arouri and Mouritsen, 2013) and the incorporation of even a small amount (1 mol%) of lysophospholipids in membranes creates instability and increased permeability in lipid bilayers (Arouri and Mouritsen, 2013).

But due to their intrinsic emulsifying properties, lysophospholipids may also directly impact the gut itself. The study by Chassaing et al. (2015) showed how dietary emulsifiers altered bacterial species composition in the gut and promoted microbiota encroachment, leading to colitis and metabolic syndrome in mice. The consequences of elevated lysophospholipid levels in cold-stored raw milk should be therefore further investigated regarding their multiple roles in both physiological and pathophysiological processes.

Altogether, the present study highlights another beneficial feature of the N_2_ gas flushing treatment. Compared to the single cold storage, N_2_ flushing better preserved the original phospholipid content and composition of raw milk for at least 7 days. The present lipidomics-based approach illustrated that N_2_ flushing is a multi-advantageous treatment, considering the bacteriological, technological, nutritional and health aspects of raw milk.

## Conclusion

Several previous reports demonstrated the benefits of a N_2_ gas flushing treatment to hinder bacterial growth in raw milk during its storage. The present study demonstrated that cold storage at 6°C for 7 days promoted hydrolysis of phospholipids (PC, SM, PE, PI, and PS) and increases in PA and in various lysophospholipid levels in raw milk. For cold-stored raw milk samples, the mass spectrometry based lipidomics investigations together with the microbiological data confirmed a major role of phospholipase C, but also revealed that other classes of phospholipases namely sphingomyelinase C, phospholipases A and D, and probably also phospholipase B (for a minor part) contributed to phospholipolysis. In contrast, the additional N_2_ gas treatment preserved initial phospholipid composition in raw milk cold- stored for at least 7 days, by hindering the production of various bacterial phospholipases, hence conferring superior quality to cold-stored raw milk.

## Author Contributions

All listed authors have made a substantial, direct and intellectual contribution to the work, and have approved the final version of the manuscript.

## Conflict of Interest Statement

The authors declare that the research was conducted in the absence of any commercial or financial relationships that could be construed as a potential conflict of interest.
